# Advances in the tea plants phenotyping using hyperspectral imaging technology

**DOI:** 10.3389/fpls.2024.1442225

**Published:** 2024-08-01

**Authors:** Baidong Luo, Hongwei Sun, Leilei Zhang, Fengnong Chen, Kaihua Wu

**Affiliations:** ^1^ College of Artificial Intelligence, Hangzhou Dianzi University, Hangzhou, China; ^2^ Key Laboratory of Specialty Agri-Products Quality and Hazard Controlling Technology of Zhejiang Province, College of Life Sciences, China Jiliang University, Hangzhou, China

**Keywords:** hyperspectral imaging, tea plants, plant phenomics, high-throughput, rapid detection, environmental stress

## Abstract

Rapid detection of plant phenotypic traits is crucial for plant breeding and cultivation. Traditional measurement methods are carried out by rich-experienced agronomists, which are time-consuming and labor-intensive. However, with the increasing demand for rapid and high-throughput testing in tea plants traits, digital breeding and smart cultivation of tea plants rely heavily on precise plant phenotypic trait measurement techniques, among which hyperspectral imaging (HSI) technology stands out for its ability to provide real-time and rich-information. In this paper, we provide a comprehensive overview of the principles of hyperspectral imaging technology, the processing methods of cubic data, and relevant algorithms in tea plant phenomics, reviewing the progress of applying hyperspectral imaging technology to obtain information on tea plant phenotypes, growth conditions, and quality indicators under environmental stress. Lastly, we discuss the challenges faced by HSI technology in the detection of tea plant phenotypic traits from different perspectives, propose possible solutions, and envision the potential development prospects of HSI technology in the digital breeding and smart cultivation of tea plants. This review aims to provide theoretical and technical support for the application of HSI technology in detecting tea plant phenotypic information, further promoting the trend of developing high quality and high yield tea leaves.

## 1 Introduction

As a popular beverage crop, tea plants are extensively cultivated in regions including Asia and Africa. For instance, over seventy million labors in China are engaged in tea-related industries in 2020, and the income of them is closely related to the yield and quality of tea product. In modern agricultural science, digital breeding and precision cultivation technologies emerged to obtain specific phenotypic traits of tea plants, and thus to promote tea production (Lu et al., 2020; Wang et al., 2023). The digital breeding mainly focuses on interpreting the genetic foundation of the tea plant phenotypic traits, while the precision cultivation technology is to unveil the regulatory network between the tea plant phenotyping and environment factors. Therefore, it can also be seen that the phenotype of tea plants is affected by the synergistic regulation of genetic material and environment. Unlike cereal crops and other horticultural plants, tea plants are typical foliar plants with unique phenotypic indices of interest. For instance, leaf area in tea plants correlates directly with yield to some extent, and the morphology of tea buds serves as a criterion in the grade evaluation of Chinese green tea. The content of polyphenols and theanine in leaves significantly influences the suitable processing methods for tea leaves and plays a critical role in the flavor and taste profile of the tea. In sum, the phenotype information of tea plants encompasses aspects such as environmental stress responses, growth status, and quality traits, characterized by rich connotations and target-oriented traits.

Compared to genetic breeding and cultivation techniques, traditional phenotypic detection in plants researches is usually performed manually, which is less efficient, highly dependent on experienced professionals, time-consuming, and labor-intensive. Especially in the determination of biochemical components in plants, wet chemistry method leads to “Phenotyping bottleneck” (Watt et al., 2020; Tao et al., 2022). In order to meet the need of high-throughput rapid detection of plant phenotyping, hyperspectral imaging technology has been paid more attention by researchers. Hyperspectral imaging (HSI) technique can be used to invert plant traits based on the changes of plant optical properties, and can collect multi-modal data of spectra and space information, which is especially suitable for simultaneous monitoring of some plant traits. Combined with machine learning and artificial intelligence technologies, HSI technology enables automated and precise detection of plant phenotypes, which has the potential to enhance the efficiency of detection, reduces artificial error, and provide more objective and accurate presentation of plant traits (Atefi et al., 2021).

In recent years, as a powerful manner for detecting plant information, hyperspectral imaging technology has been extensively applied in tea plant phenotyping researches, involving environmental resistant, physiological, and biochemical traits. However, many challenges are faced by HSI technology to further promote the application in digital breeding and precision cultivation research. For example, it is necessary to deeply understand the relationship between tea plant phenotypic traits and hyperspectral information, and comprehensively analyze its feasibility and limitations for phenotyping. Hence, we elucidate the principles of hyperspectral imaging technology and summarize typical workflows and related algorithmic techniques for hyperspectral data analysis. Based on these previous studies, we categorize the application of hyperspectral imaging technology in tea plant phenotyping into environmental stress responses, growth status, and quality traits. Simultaneously, we discuss the current challenges faced when using HSI technology in the detection of tea plant phenotypic information and potential future trends. Our aim is to comprehensively summarize the current state of tea plant phenotypic information detection researches using hyperspectral imaging technology and provide in-depth commentary and suggestions on the current predicaments in the analysis of tea plant phenotyping.

## 2 Principles of hyperspectral imaging technology for tea plant phenotypic detection

**Figure 1:**
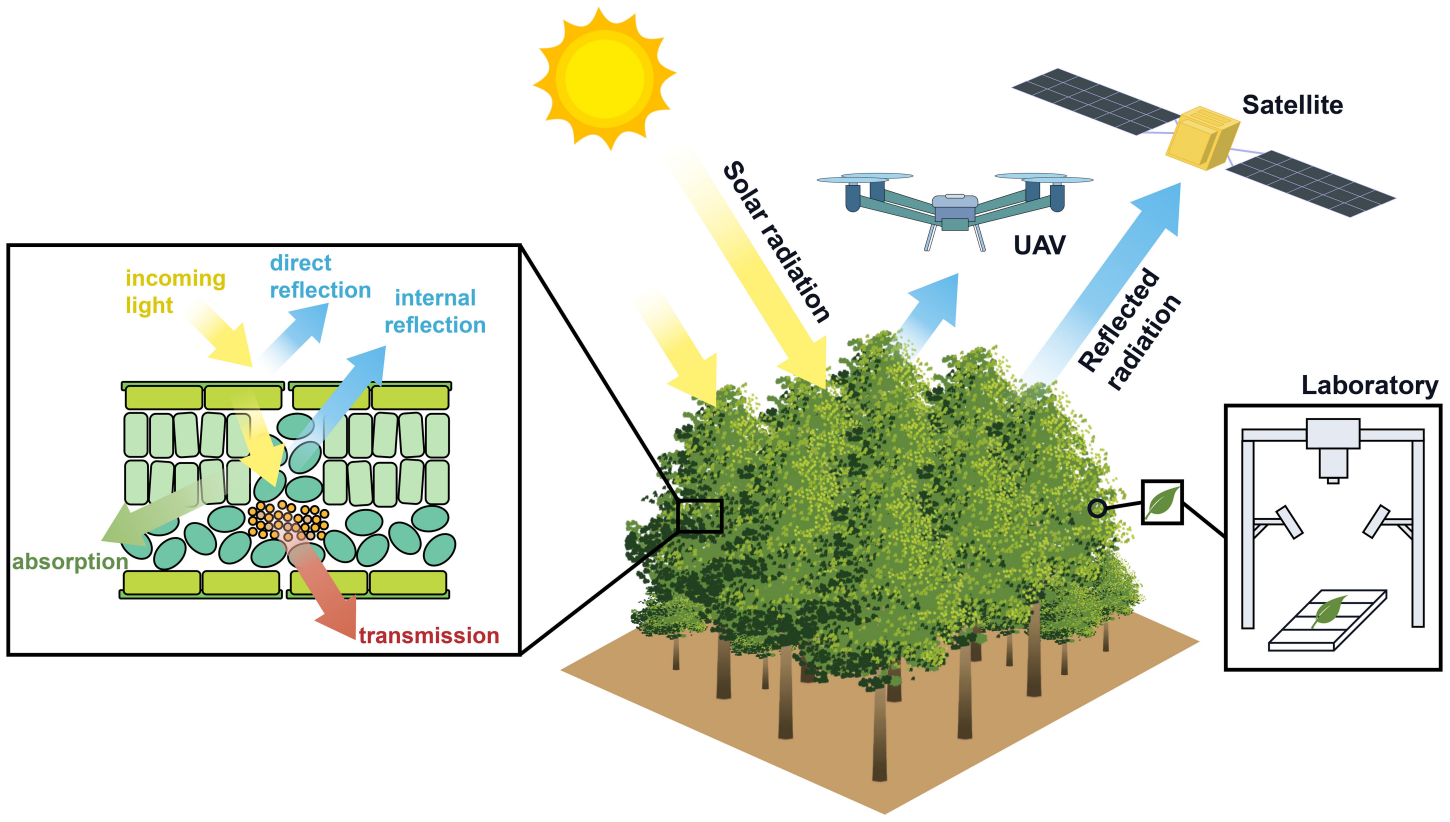
Multi-scale hyperspectral data measurement scheme.

Hyperspectral imaging technology combines spectroscopy and computer vision, which is able to measure the optical absorption, scattering, and reflectance properties of plant tissues and organs across a wide variety of wavelengths, to perform quantitative analyses and qualitative assessments for key agronomic traits such as the growth status, yield, and quality of tea plants. In the visible to near-infrared spectral wavelength (400-2500nm), this technology captures the molecular vibration information of plant biochemical components with hydrogen-containing groups, like chlorophyll and other photosynthetic pigments (Slaton et al., 2001; Tang et al., 2024). In addition, hyperspectral imaging technology also has the ability to monitor the spatial distribution of plant phenotypic traits at different scales, from cells, organs, individuals to populations, as shown in **Figure 1** (Mahlein, 2016; Kuswidiyanto et al., 2022). Therefore, hyperspectral data is always stored in the format of a multi-dimensional cube, as its spectral dimension records chemical component content in plant leaf, while the spatial dimension details the distribution of various compositional contents of canopy, or the morphological structures of tea plant and epidemic range of pests and diseases. For instance, by utilizing visual representations of moisture distribution in tea leaves, one can observe the water content at each pixel level in a more intuitive and comprehensive manner, thereby offering an innovative approach for assessing irrigation needs of tea plants (Sun et al., 2019).

In the plant phenomics research field, the principles of plant phenotyping using hyperspectral imaging technology can be divided into direct and indirect manners (Zhang et al., 2020). In the study of detecting the content of water and polyphenols in leaves by hyperspectral imaging technology, since water and polyphenols have strong absorption peaks in the near-infrared spectral band, a mathematical model between the absorbance of tea leaves and the content of water and polyphenols can be directly constructed, which belongs to direct detection methods. In plant pest detection studies, plant leaves often appear different forms of disease spots, necrotic or wilted areas after being infected by germs, and the content and activity of pigments would be reduced, resulting in an increase in the reflectivity of the visible region, while the red edge (670-730nm) moves to the shortwave direction (Bai et al., 2020). In these process of plant infestation by pests and diseases, the spectral response is related to the symptoms on plant pigment, water, leaf area and other biochemical substances caused by diseases and pests, which belongs to indirect detection methods. Compared to direct detection, the precision of indirect detection is commonly susceptible to variations influenced by plant species, age, and even the cultivation environment, resulted in necessary to recalibrate the detection model in a period of time. However, this method can reflect the relationship between different traits of plants, as well as the influencing factors and evolution rules.

## 3 The workflow of HSI application in tea plant phenotypic analysis

**Figure 2:**
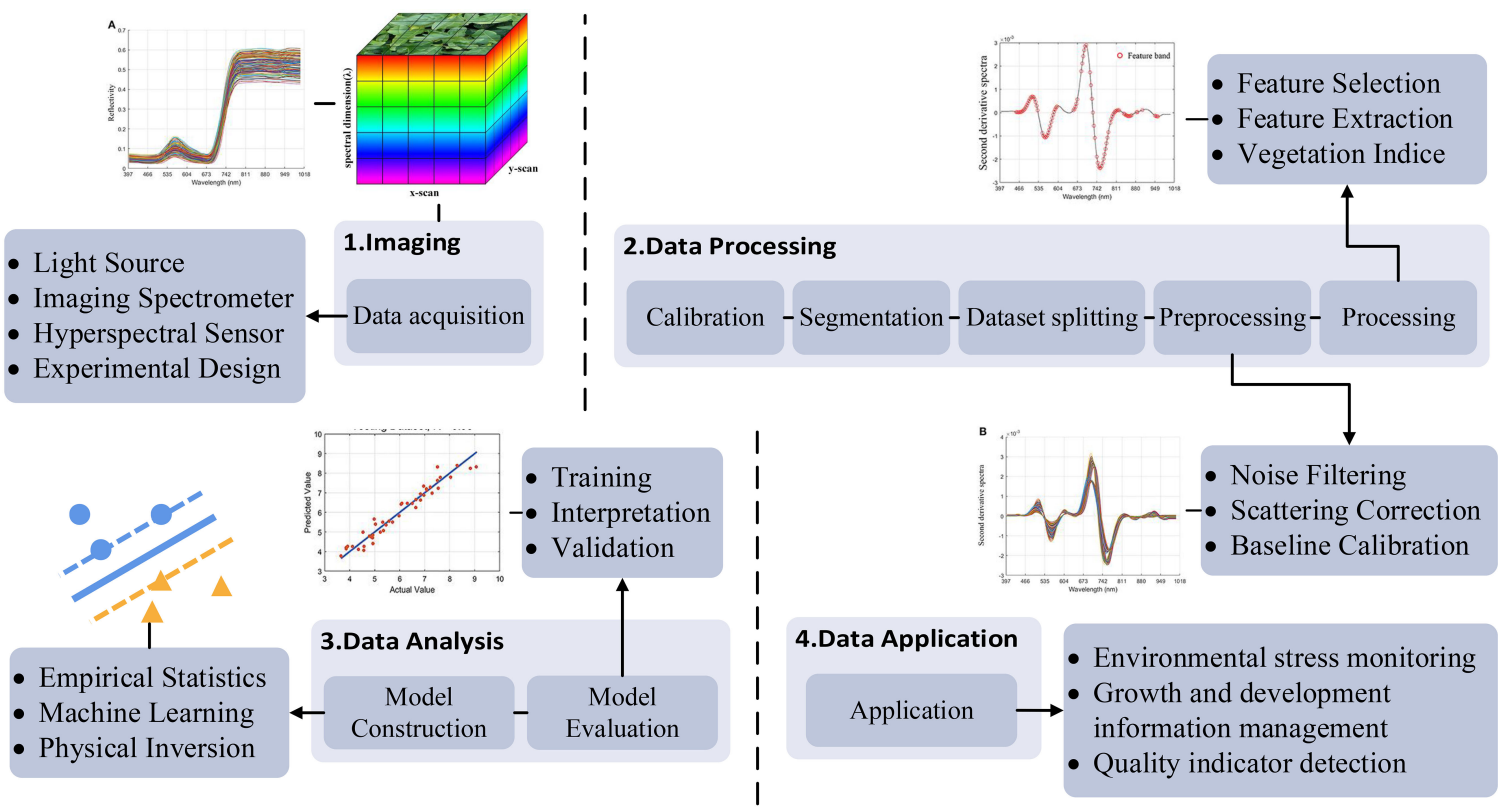
Workflow of hyperspectral data processing.

No uniform standard workflow for hyperspectral data processing in tea plants has been established. However, drawing upon a summary of previous researches in detecting tea plant phenotypic information using hyperspectral imaging technology, the entire data processing workflow can generally be divided into four steps: data acquisition, data preprocessing, data analysis, and data application (**Figure 2**) (Li et al., 2013; Paulus and Mahlein, 2020).

### 3.1 Hyperspectral data acquisition

Hyperspectral imaging systems are usually deployed on diverse platforms such as satellites, airplanes, unmanned aerial vehicles (UAVs) and ground-based fixed equipment, which is typically composed of components like light sources, optical lens, imaging spectrometers, photoelectric detector, and computer data processing systems (Lu et al., 2020). In the process of capturing optical characteristic information of tea plants, samples are illuminated by either natural or artificial light sources (such as halogen lamps), and the optical lens gathers the reflected light from the samples. This reflected light is split into different wavelengths by a diffraction grating element within the spectrometer, and photoelectric detector subsequently transform these light intensity into electrical signals. Finally, computer data processing systems acquire and process these electrical signals, transform them into digital signals, and form a spectrum of target plant (Mishra et al., 2017).

**Figure 3:**
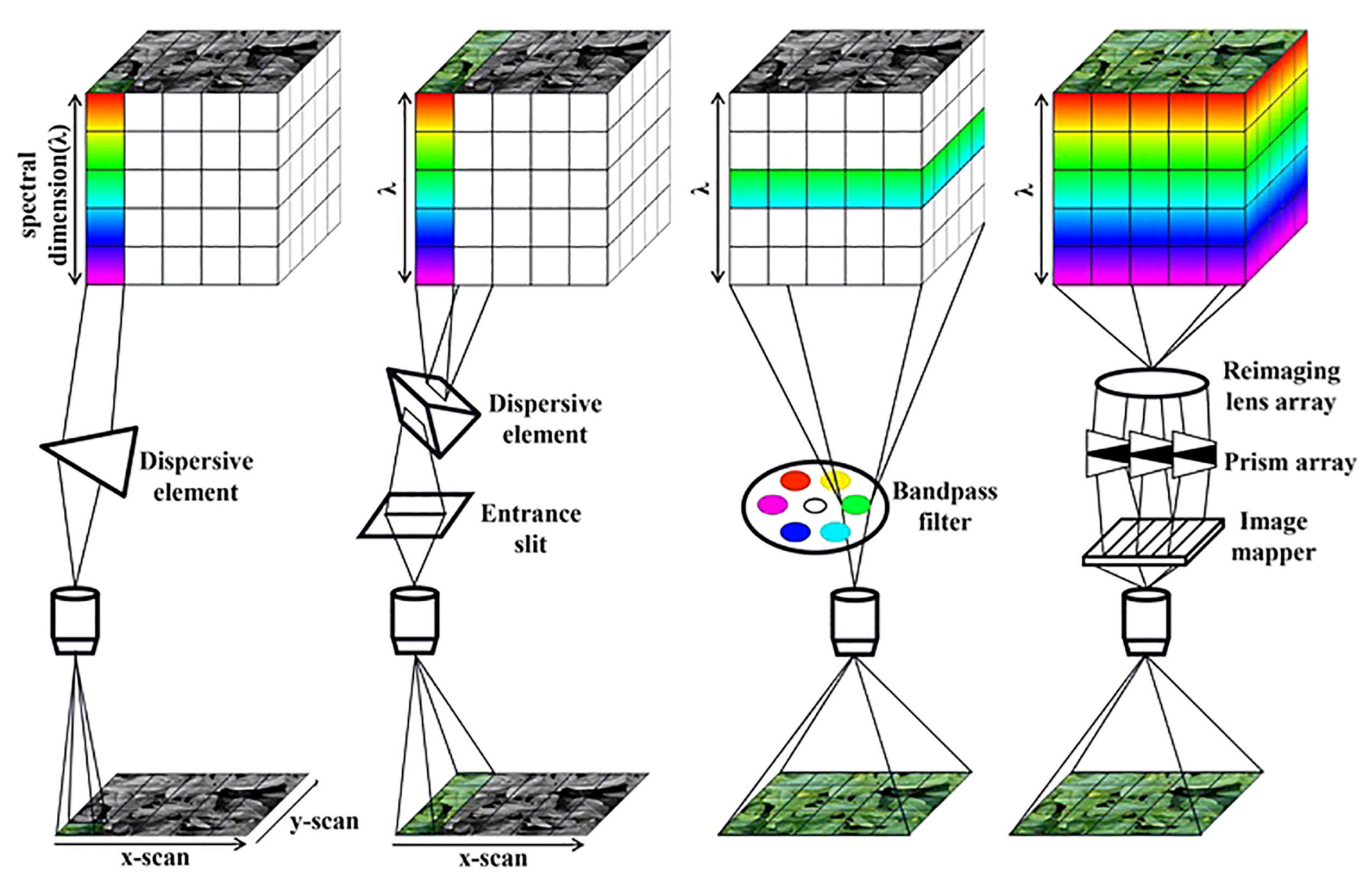
Four modes of hyperspectral data acquisition.

There are mainly four hyperspectral data collection methods: point scanning, line scanning, area scanning, and snapshot (**Figure 3**). Point scanning (whisk-broom) acquires spectral information pixel by pixel, providing high hyperspectral resolution, its slow speed requires a highly stable platform to avoid image blurring and distortion. Line scanning (push-broom), currently the most commonly used scanning method, captures one row of pixels at a time, balancing spatial and spectral resolution, but care should be taken to minimize motion artifacts during the acquisition process. Area scanning (staring) uses bandpass filters to capture image data for individual bands, scanning band by band to construct a hyperspectral data cube, providing high spatial resolution images, but the scene must be kept stationary during acquisition to ensure the accuracy of the localization and spectral information. Snapshot technology can simultaneously capture spatial and spectral information of the entire scene. A single snapshot represents a perspective projection of the data cube, from which its three-dimensional structure can be reconstructed. However, as this technique is at an early stage of development, the captured images often exhibit comparatively low spatial and spectral resolution (Hagen and Kudenov, 2013). Based on their respective detection principles, each mode has its own set of advantages and weaknesses for practical application. With an in-depth understanding of the basic principles of each imaging modes, we are able to flexibly choose the appropriate acquisition manner in various acquisition environments to maximize the imaging requirements and improve the data acquisition efficiency. In this case, different data collection methods also make it difficult to standardize data, so it is necessary to build a hyperspectral phenotyping data sharing platform, which not only facilitates the global exchange of raw data and processing methods, so as to maintain the consistency of data (Xu et al., 2020; Sun et al., 2022). Despite continuous advancements in hyperspectral imaging technology, the high resolution it offers comes with significant costs that may detract from its cost-effectiveness compared to other spectral sensing techniques. Nevertheless, as optical technologies continue to evolve, these economic barriers are expected to be progressively mitigated, thereby fostering the mainstream adoption of hyperspectral imaging in precision agriculture, environmental monitoring, and beyond.

### 3.2 Hyperspectral data preprocessing

Before the spectral domain analysis of hyperspectral data, it is necessary to segment the image data in the spatial domain to extract the spectral and spatial characteristics of the target. Image segmentation aims to effectively differentiate various substances or objects within an image, dividing them into mutually independent regions, with each region consisting of a collection of pixels that share certain similar characteristics and attributes. For the detection of plant phenotypic information, such operation is to generate masks to accurately demarcate regions of interest (ROI), thus eliminating irrelevant or even possible interference information (ElMasry et al., 2009). Common strategies for image segmentation include threshold segmentation, morphological processing, edge segmentation and spectral image segmentation (Tripathi et al., 2012; Wu and Sun, 2013). Typically, before implementing hyperspectral image segmentation, a single band sub-image with the greatest contrast against the background is selected and segmented to serve as a mask for excluding background pixels (Wang et al., 2020d). In the case of threshold segmentation, an appropriate threshold is set to convert a grayscale image into a binary image mask, to extract hyperspectral data of target object based on its position in the image.

When measuring plant spectral data using a hyperspectral imaging system, interference noise caused by variations from the collection instrument, light source, and environmental conditions is often encountered (Mishra et al., 2020; Çetin and Yıldız, 2022). Depending on the characteristics, noise typically manifests as continuous disturbances or transient spikes in the data. The Savitzky-Golay (S-G) filter algorithm (Steinier et al., 1972), widely used for smoothing, effectively removes the noise while preserving the overall trend of the data by fitting a polynomial to the input signal, while the limited effects in removing low-frequency noise from spectral data. During practical measurement, the appearance of multi-source noise in spectral data may be accompanied by baseline drift (Zhang and Wang, 2023). Derivative algorithms can realize the correction of baseline effects on the basis of removing background interference and reduce the impact of baseline drift. Nevertheless, directly applying a high-order derivative process to spectral data with a low original signal-to-noise ratio might amplify the noise present in the spectrum (Yan et al., 2000; Chu et al., 2004). Therefore, for spectra with low signal-to-noise ratios, the derivative algorithm needs to be used with caution, and noise smoothing or other preprocessing steps may need to be applied before proceeding to minimize potential noise effects. Moreover, the complex tissue structure and surface microfeatures of tea plants can lead to non-uniform scattering of light at different locations, resulting in various scattering effects. Standard Normal Variate Transformation (SNV) (Barnes et al., 1989) and Multiplicative Scatter Correction (MSC) (Wu et al., 2019) are commonly used to correct scattering effects. The MSC algorithm adjusts scattering effects in spectra by comparing the ratio of sample spectra to reference spectra (Geladi et al., 1985), while the SNV algorithm performs mean subtraction and standard deviation division at each wavelength to transform the spectral data into a standard normal distribution. Both algorithms can eliminate scattering effects and spectral differences caused by baseline drift, reducing noise interference encountered during derivative algorithm processing. To showcase the characteristics of commonly used preprocessing algorithms in hyperspectral imaging techniques for tea plant phenotypic analysis, **Table 1** provides a detailed description of the operating mechanism of each preprocessing technique. To further enhance the effectiveness of spectral data preprocessing, a combination of various preprocessing techniques or structural improvements and parameter optimizations of classical algorithms is commonly employed. Additionally, machine learning models are increasingly applied to spectral preprocessing. For instance, autoencoders (AE) utilize self-supervised learning methods to map noisy inputs to latent variable space, reconstructing outputs that closely approximate the original noise-free data, thus achieving automatic denoising (Zhang et al., 2020). The application of these methods not only improves the accuracy and efficiency of spectral data modeling but also lays a solid foundation for conducting more complex analyses and applications.

**Table 1:** Common preprocessing algorithms used in hyperspectral imaging technology for tea plant phenotypic information detection.

Common preprocessing algorithms		Functions	References
Algorithms	Abbreviations		
Normalization	-	Elimination of redundant information, resulting in better interpretability and comparability of data	(Chu et al., 2004)
Standardization	-		
Mean Centering	-		
Orthogonal signal correction	OSC	Filtering out irrelevant information and retaining the main information in the original spectrum	(Wold et al., 1998)
Savitzky-Golay smoothing	S-G	Preserving the essential information and the morphology of the signal while reducing random high-frequency noise in the spectral data	(Steinier et al., 1972)
Moving average smoothing	-		
Standard normal variate transformation	SNV	Elimination of non-uniform scattering due to heterogeneity of tea plant surfaces	(Barnes et al., 1989)
Multiplicative scatter correction	MSC		(Wu et al., 2019)
Derivative	1st Der,2nd Der	Eliminating baseline effects and removing background interference for improved resolution and sensitivity	(Chu et al., 2004)
De-trending	DT	Eliminating baseline drift, baseline tilt and curvature	(Wold et al., 1998)

### 3.3 Hyperspectral data processing

The huge amount of hyperspectral data often leads to the complexity of the calculation, which makes it difficult to find really useful information. It is imperative to select the most informative features from a multitude of bands and remove redundant or unnecessary data (Cheng et al., 2022; Omia et al., 2023). Therefore, prior to establishing relational model between plant phenotypic trait and hyperspectral data, it is often necessary to perform dimensionality reduction operations such as feature selection or feature extraction to address curse of dimensionality of hyperspectral data. Feature selection is a method of filtering out the most representative subset of features from the original feature set. The aim is to preserve information most relevant to the research objective and eliminate other unrelated features to reduce the impact of redundancy and noise (Adao et al., 2017). In the process of analyzing tea plant phenotypic information, Competitive Adaptive Reweighted Sampling (CARS) and Uninformative Variable Elimination (UVE) are two commonly used feature selection methods. The CARS algorithm draws on the “survival of the fittest” principle and calculates the competitiveness score of features based on their inter-correlation within a subset. Li et al. used the CARS algorithm to select 19 representative bands from a dataset of 700 bands, and the corresponding CARS-PLS model reduces the RMSECV by 0.0433 in terms of detection effectiveness compared to the traditional PLS model (Li et al., 2009). The UVE algorithm, on the other hand, selects features with high information gain or chi-square statistic between the selected target variable and the features as the initial selection set, and progressively removes the uninformative variables that do not have a significant impact on the classification performance (Centner et al., 1996). Feature extraction is the process of mapping original features onto a new feature space through a certain transformation method and then selecting key features that can describe essential characteristics of the original data. The Successive Projections Algorithm (SPA) (Milanez et al., 2017) is a widely used feature extraction method that sequentially selects projection directions that maximally retain key characteristics of the original data, mapping high-dimensional data into a lower-dimensional subspace to achieve data dimensionality reduction. In the study of post-harvest fresh tea leaf quality indicators, the SPA-MLR model constructed via SPA significantly outperformed other models in optimizing model performance, demonstrating that the algorithm improves prediction accuracy while simplifying the modeling process (Wang et al., 2020b). Above all, these dimensionality reduction algorithms are data-driven, and it is difficult to explain the physical meaning behind them. And in fact, hyperspectral imaging equipment is facing the dilemma of high cost. In response to this challenge, researchers have ventured into the deployment of multispectral imaging technology, which is more economically viable (Tran, 2001; Xu et al., 2023). This technology typically involves the acquisition of a finite number (usually not exceeding ten) of discrete broadband (50-200nm) images for target objects. By amalgamating hyperspectral data with theoretical analysis to pinpoint wavelength variables associated with specific plant phenotype indices, multispectral technology emerges as an efficacious alternative, especially in designing portable instruments targeted at specific detection objectives (Wang et al., 2023). Future developments in portable multispectral instruments not only have the potential to lower the application costs of the technology but also to broaden the applicability of hyperspectral technology in agricultural production practices, thereby enhancing its viability and economic benefits in real-world production environments (Yang et al., 2021; Saric et al., 2022).

Moreover, in the study of satellite remote sensing and UAV observation, the construction of vegetation indices is also a common method for processing hyperspectral data. Typically formulated through combinations of the differences or ratios of two or more spectral band variables, vegetation indices serve to represent one or multiple parameters indicative of vegetation status (Xue and Su, 2017; He et al., 2023). Typical multispectral vegetation indices are primarily composed of red and near-infrared spectral bands, as the red band reflects chlorophyll content of vegetation, while the near-infrared band is closely related to the structural characteristics of plant leaves. Such indices are primarily employed in performing vegetation monitoring tasks and are capable of mitigating the influence of background interference to some extent. The advent of hyperspectral technology broadens the observable spectral range and offers more refined spectral bands. This enables the hyperspectral vegetation indices to be used for more detailed and precise monitoring of general plant phenotypes on the basis of the original, and to be able to estimate and invert more relevant phenotypic features (Verrelst et al., 2015; He et al., 2023). For instance, the utilization of hyperspectral vegetation indices allows differentiation between vegetation individuals and populations under various stresses, such as water stress and nutrient deficiency. The high-precision spectral data thus provided carry unparalleled scientific value for grasping the health status of vegetation, growth dynamics, and even adaptive studies in response to environmental changes (Fu et al., 2021). **Table 2** lists some commonly used hyperspectral vegetation indices in the research for detecting phenotypic information in tea plants.

**Table 2:** Commonly used hyperspectral vegetation indices in the study of tea plant phenotypic information detection.

Vegetation Indices		Characteristics and Functions	Definition	Reference
Structure (LAI, Green biomass, etc.)				
Greenness Index	GI	Estimate biochemical constituents and LAI at leaf and canopy levels	$R_{554}/R_{677}$	(Zarco-Tejada et al., 2005)
Improved Soil Adjusted Vegetation Index	MSAVI	A more sensitive indicator of vegetation amount than SAVI at canopy level	$0.5[2R_{800}+1-({\left(2R_{800}+1\right)}^{2}$ $-8\left(R_{800}-R_{670}\right){)}^{1/2}]$	(Qi et al., 1994)
Narrow Band Normalized Difference Vegetation Index	NBNDVI	Responds to change in the amount of green biomass and more efficiently in vegetation with low to moderate density	$\left(R_{874}-R_{1225})/(R_{874}+R_{1225}\right)$	(Rouse et al., 1974)
Hyperspectral Perpendicular VI	${\text{PVI}}_{\text{hyp}}$	More efficiently quantify the low amount of vegetation by minimizing soil background influence on vegetation spectrum	$(R_{1148}-aR_{807}-b)/{\left(1+a^{2}\right)}^{1/2},$ where a = 1.17,b = 3.37	(Schlerf et al., 2005)
Normalization or Standard of the LAIDI	sLAIDI	Sensitive to LAI variation at canopy level with a saturation point >8	$\text{S}(R_{1050}-R_{1250})/(R_{1050}+R_{1250}),$ where S=5	(Delalieux et al., 2008)
Pigments (Chls, Cars)				
Leaf Chlorophyll Index	LCI	Estimate Chl content in higher plants, sensitive to variation in reflectance caused by Chl absorption	$\left(R_{850}-R_{710})/(R_{850}+R_{680}\right)$	(Datt, 1999)
Modified Chlorophyll Absorption in Reflectance Index	MCARI	Respond to Chl variation and estimate Chl absorption	$\left[(R_{701}-R_{671})-0.2(R_{701}+R_{549})\right]$ $\times \left(R_{701}/R_{671}\right)$	(Daughtry et al., 2000)
Ratio Analysis of Reflectance Spectra	RARS	Estimate carotenoid pigment contents in foliage	$R_{760}/R_{500}$	(Chappelle et al., 1992)
Triangle chlorophyll vegetation index	TCI	Quantify chlorophyll content of tallgrass at leaf and canopy levels	$[(R_{800}+1.5\times R_{550})-R_{675}]/$ $\left(R_{800}-R_{700}\right)$	(Gao, 2006)
Vogelmann red edge index	VOG	Sensitive to the combined effects of foliage chlorophyll concentration, canopy leaf area, and water content	$R_{740}/R_{720}$	(Vogelmann et al., 1993)
Water				
Leaf Water VI 1	LWVI-1	Estimate leaf water content, an NDWI variant	$\left(R_{1094}-R_{893})/(R_{1094}+R_{893}\right)$	(Galvao et al., 2005)
ND Water Index	NDWI	Improving the accuracy in retrieving the vegetation water content at both leaf and canopy levels	$\left(R_{860}-R_{1240})/(R_{860}+R_{1240}\right)$	(Gao, 1996)
Shortwave Infrared Water Stress Index	SIWSI	Estimate leaf or canopy water stress, especially in the semiarid environment	$\left(R_{860}-R_{1640})/(R_{860}+R_{1640}\right)$	(Fensholt and Sandholt, 2003)
Water Index	WI	Quantify relative water content at leaf level	$R_{900}/R_{970}$	(Peñuelas et al., 1997)
Stress				
Red-Edge Vegetation Stress Index	RVSI	Assess vegetation community stress at canopy level	$\left[(R_{712}+R_{752}\right)]/2-R_{732}$	(Merton and Huntington, 1999)

### 3.4 Construction of phenotypic information detection models

**Figure 4:**
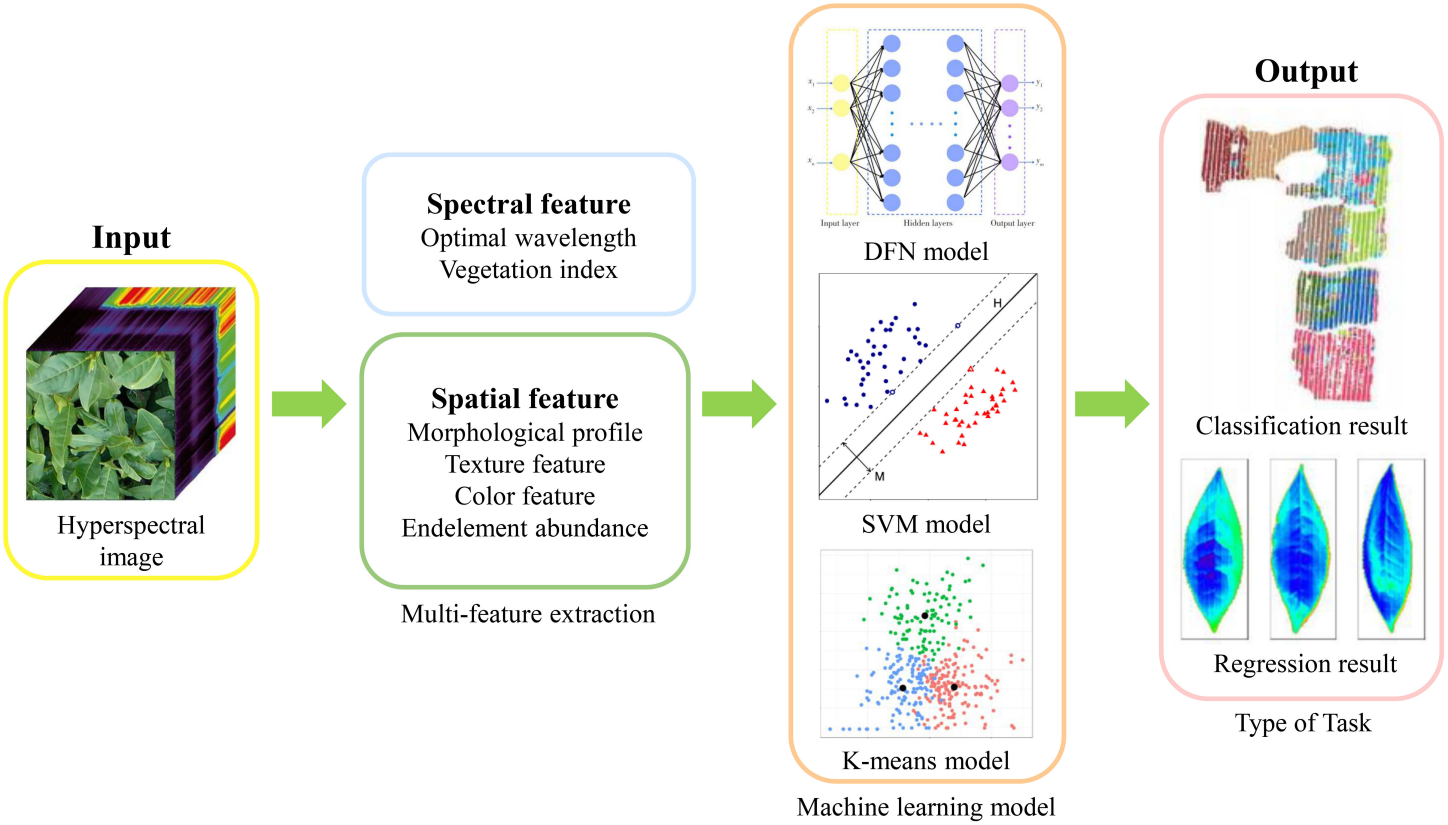
Workflow for constructing the phenotypic information detection model.

The most critical step in the workflow of tea plants phenotyping using hyperspectral technology is the construction of math models between plant traits and plant optical characteristics. As illustrated in **Figure 4**, a model is established using machine learning techniques, with measured spectral characteristics, spatial features, or fused features as independent variables, and actual measured phenotypic parameters of tea plants as dependent variables (Ang and Seng, 2021).

During model construction, we will involve two types of univariate statistical models and multivariate statistical models. The former focuses on the statistical analysis of a single variable to reveal characteristics of the data. In phenotypic studies of tea plants, univariate statistical techniques typically involve the creation of spectral feature variables such as vegetation indices variables and spectral position variables, etc., which reflect phenotypic characteristics of the tea plants through statistical processing of specific bands (Sonobe et al., 2018b; Cao et al., 2022b). According to Wu et al. the constructed vegetation index variable ${\text{VI}}_{4}$ ( ${\text{VI}}_{4}=SD_{r}/SD_{y}$ ) not only had a correlation coefficient as high as 0.799 with the nitrogen content of tea tree leaves, but also the index model constructed using ${\text{VI}}_{4}$ had the lowest RMSE of 0.1029, which showed an excellent fitting effect (Wu et al., 2018). On the other hand, statistical analysis methods rely on the entire spectral range or selected feature bands, aiming to comprehensively recognize patterns hidden amongst multiple variables, thereby facilitating an in-depth understanding of the overall data characteristics. Regression algorithms, one class of statistical analysis methods, are often used in plant phenotyping task, to detect continuous plant traits such as plant height, chlorophyll content. The most commonly used multivariate statistical models in current research are the Multiple Linear Regression (MLR) model (Huang et al., 2020) and the Partial Least Squares Regression (PLSR) model (Cao et al., 2022a). The PLSR model, in particular, is designed with the correlation among independent variables in mind and has the capacity to transform variables in the original spectral data that exhibit multicollinearity into a small number of independent latent variables. Therefore, PLSR models typically exhibit superior predictive efficacy, thus it is regarded as a fundamental method for hyperspectral data analysis. Besides regression task, classification and clustering are also common phenotypic information detection models (Plaza et al., 2009; Layne et al., 2020). The former aims to predict discrete category labels or to distinguish image pixels, such as tea plant breeds identification, environmental stress diagnosis, and disease and pest regions detection. The latter can be used to find the internal structure of a data set. Tea plant samples can be divided into autonomous groups by applying clustering techniques [e.g., K-means (Shan et al., 2018)], which allows for higher similarity between data within the same category than between data belonging to other categories (Davis et al., 2020). Clustering algorithms can also be employed in image segmentation, as it can proficiently discern and identify objects and shapes within images (Zhang and Xu, 2018; Nikbakhsh et al., 2020).

Deep learning, as a new branch of machine learning, focuses on the architecture and function of neural networks, particularly deep neural networks. In recent years, it has demonstrated outstanding performance in applications such as agricultural pest and disease detection (Golhani et al., 2018; Xiao et al., 2022), and stress monitoring (Singh et al., 2018). Compared to conventional machine learning approaches, deep learning models autonomously discover latent patterns and features within data, obviating the need for manual feature engineering and selection. Deep neural networks consist of multiple hidden layers, each transforming input data, enabling deeper networks to capture more intricate features (LeCun et al., 2015; Zhao et al., 2022b). This capability makes deep learning especially suitable for handling high-dimensional and large-scale complex datasets. Currently, some deep learning models have been applied to phenotypic studies of tea plants. For instance, models based on convolutional neural networks (CNN) exhibit superior overall performance in quantifying low temperature stress in tea leaves (Mao et al., 2023a), compared to traditional machine learning methods. As deep learning technologies advance, the introduction of generative models and the proliferation of large-scale models in the processing of agricultural hyperspectral data deserve particular attention. Through generative models, effective data augmentation and synthesis, data dimensionality reduction, anomaly detection, and spectral unmixing are facilitated, thereby opening new avenues in hyperspectral data processing (Bhatt and Joshi, 2020; Hu et al., 2022; Guerri et al., 2024). These advancements not only enhance the performance of existing methods but also propel hyperspectral data processing towards greater intelligence and efficiency.

## 4 Application of hyperspectral imaging technology in tea plant phenotyping

With an overview of the researches published recently, we reviewed the application of hyperspectral imaging technology in tea plant phenotyping from three aspects: environmental stress diagnosis, growth state monitor, and quality indicators estimation. Hyperspectral imaging technology could provide high-throughput comprehensive technical support and data source for tea plant phenotyping. In **Table 3**, we have summarized the application of hyperspectral imaging technology to the phenotypic traits of tea plants.

**Table 3:** Examples of the application of hyperspectral imaging technology in the detection of tea plant phenotypic information.

Phenotypic Trait	Domain	Data preprocessing	Data processing	Data analysis	Optimal performance	Refs
Quality indicator detection						
Polyphenols	Laboratory	1st Der, Smoothing, Normalization	SPA	MLR, LS-SVM	${\text{Rp}}^{2}$ (EGC) = 0.918 ${\text{Rp}}^{2}$ (EGCG) = 0.918 ${\text{Rp}}^{2}$ (EC) = 0.918 ${\text{Rp}}^{2}$ (ECG) = 0.918	(Dutta et al., 2015; Zhang, 2017; Tu et al., 2018)
	Field	WD, CR, SNV, 1st Der, 2nd Der	MNF, PCA, ICA	MLC, MDC, ANN, SVM, PLSR, BPANN	${\text{R}}_{\text{CV}}$ = 0.9322 ${\text{RMSE}}_{\text{CV}}$ = 0.9036	
		SR, NR, FDR	SDA, PCA	SMLR, PLSR	${\text{R}}^{2}$ = 0.81 RMSE = 1.39	
Amino acids	Field	WD, CR, SNV,1st Der,2nd Der	MNF, PCA, ICA	MLC, MDC, ANN, SVM, PLSR, BPANN	${\text{R}}_{\text{CV}}$ = 0.9322 ${\text{RMSE}}_{\text{CV}}$ = 0.9036	(Tu et al., 2018)
Environmental stress monitoring						
Heavy metal stress	Laboratory	BC, DT, SNV, MSC	SPA, CARS, CARS-SPA	MLR, LS-SVM, PLS	${\text{R}}^{2}$ (Chl) = 0.691 ${\text{R}}^{2}$ (ASA) = 0.596 ${\text{R}}^{2}$ (GSH) = 0.646 ${\text{R}}^{2}$ (SP) = 0.624	(Jin, 2019)
Drought	Laboratory	SG, 1st Der, 2nd Der, MSC	SPA, CARS, UVE	RF, SVM, PLSR	Rcal = 0.97 Rp = 0.95 RPD = 4.28	(Chen et al., 2021, 2022)
		SG, 1st Der, 2nd Der, MSC, SNV	SPA, CARS, UVE	RF, SVM, PLSR	${\text{R}}^{2}\;$ = 0.77 RMSE = 0.073 MAPE = 0.16	
Low temperatures	Laboratory	SG, 1st Der, MSC	SPA, CARS, UVE	RF, SVM, PLS, BP, CNN, LSTM	${\text{R}}^{2}$ = 0.890 ${\text{RMSE}}_{\text{P}}$ = 0.325 RPD = 2.904	(Mao et al., 2023a, 2023)
		SG, 1st Der, MSC	SPA, UVE	SVM, PLS, CNN	${\text{Rp}}^{2}$ = 0.812 ${\text{RMSE}}_{\text{P}}$ = 0.008	
Tea green leafhopper	Laboratory	-	CWA, Spectral derivative features, Continuum removal features	K-means, SVM, FLDA, RF, SVM	OA = 90.69%	(Cui et al., 2021; Zhao et al., 2022a)
	Field	-	SPA, Spectral derivative features, Continuum removal features, Vegetation indices	KNN, RF, Fisher	OA >= 98%	
Anthracnose	Laboratory	SG, 2nd Der, SNV	SPA, PLS-DA	SVM, ELM	${\text{RMSE}}_{\text{P}}$ = 0.2874 Accuracy = 95.77%	(Yuan et al., 2019; Bing et al., 2021; Cui et al., 2021; Zhao et al., 2022a)
		-	Feature variables, Vegetation indices	ISODATA	OA = 98% Kappa = 0.96	
		-	CWA, Spectral derivative features, Continuum removal features	K-means, SVM, FLDA, RF, SVM	OA = 90.69%	
	Field	-	SPA, Spectral derivative features, Continuum removal features, Vegetation indices	KNN, RF, Fisher	OA >= 98%	
White star Disease	Laboratory	SG, 2nd Der, SNV	SPA, PLS-DA	SVM, ELM	${\text{RMSE}}_{\text{P}}$ = 0.2874 Accuracy = 95.77%	(Bing et al., 2021)
Growth and development information management						
Water	Laboratory	-	RF, SPA	PLSR, LS-SVR	Rp2 = 0.956 ${\text{RMSE}}_{\text{P}}$ = 0.027	(Sun et al., 2019; Wei et al., 2019; Wang et al., 2020b)
		SG, OSC, MSC, DT	SPA, CARS, SPA-SR, CARS-SR	MLR	Rp2 = 0.8631 ${\text{RMSE}}_{\text{P}}$ = 0.0163	
		-	SPA, CARS	MLR, PLSR	Rp = 0.8543, 0.9357,0.8188	
Chlorophyll	Laboratory	-	-	Vegetation indices	R = 0.8323 RMSE = 8.601	(Zhao et al., 2011; Sonobe et al., 2018a; Wang et al., 2019; Yamashita et al., 2020)
		1st Der, CR, SNV, MSC, DT	-	RF, SVM, Cubist, SGB, KELM	RPD(N) = 0.0163 RPD(Chl) = 0.0163	
		1st Der, SNV, MSC	2nd Der, RC	PLSR	Rp = 0.9322 ${\text{RMSE}}_{\text{P}}$ = 0.9036	
		-	GA	RF, DBN, ELM, SVM	RMSE = 0.36 ± 0.08	
Carotenoid	Laboratory	1st Der, SNV, MSC	2nd Der, RC	PLSR	Rp = 0.9322 ${\text{RMSE}}_{\text{P}}$ = 0.9036	(Sonobe et al., 2018a; Wang et al., 2019)
		-	GA	RF, DBN, ELM, SVM	RMSE = 0.36 ± 0.08	
Nitrogen	Laboratory	SNV	-	PLS-DA, LS-SVM, PLSR	CCR = 92% Rp = 0.924 ${\text{RMSE}}_{\text{P}}$ = 0.209	(Wang et al., 2018; Wu et al., 2018; Yamashita et al., 2020; Wang et al., 2020b, 2020)
		-	PCA, GLGCM	SVM, ELM	CCR = 100%	
		-	SPA, CARS	MLR, PLSR	Rp = 0.8543, 0.9357,0.8188	
		1st Der, CR, SNV, MSC, DT	-	RF, SVM, Cubist, SGB, KELM	RPD(N) = 0.0163 RPD(Chl) = 0.0163	
	Field	-	Feature variables	Regression model	${\text{R}}^{2}$ (N) = 0.8303 RMSE(N) = 0.1029 ${\text{R}}^{2}$ (LAI) = 0.9 RMSE(LAI) = 0.0876	
Phosphorus, Potassium	Laboratory	SNV, MSC, 1st Der, 2nd Der, Normalization	SPA, RC	PLSR, MLR	Rp = 0.9168 ${\text{RMSE}}_{\text{P}}$ = 0.4941	(Wang et al., 2020a)
Leaf Area Index	Field	-	Feature variables	Regression model	${\text{R}}^{2}$ (N) = 0.8303 RMSE(N) = 0.1029 ${\text{R}}^{2}$ (LAI) = 0.9 RMSE(LAI) = 0.0876	(Wu et al., 2018)
Growing status	Field	-	Spectral band, Spectral derivative features, Continuum removal features, Vegetation indices	FLDA, RF, GA-SVM	OA = 98% Kappa = 0.97	(Zhang et al., 2019)

### 4.1 Environmental stress diagnosis

The phenotype of plants is influenced by a combination of genetic and environmental factors. Hyperspectral imaging technology has been widely used in studying the phenotype of tea plants under environmental stress, including both biotic and abiotic stress conditions (Sun et al., 2021; Sanaeifar et al., 2023). Physiological changes triggered by these conditions can significantly affect the growth process of tea plants. Accurate monitoring and assessment of the degree of environmental stress on tea plants through hyperspectral imaging technology can not only take timely and reasonable measures to alleviate the negative impact of stress on tea plant growth, but also help the selection and breeding of resistant tea plant varieties, which in turn promotes the enhancement of tea yield and quality.

#### 4.1.1 Biotic stress

**Figure 5:**
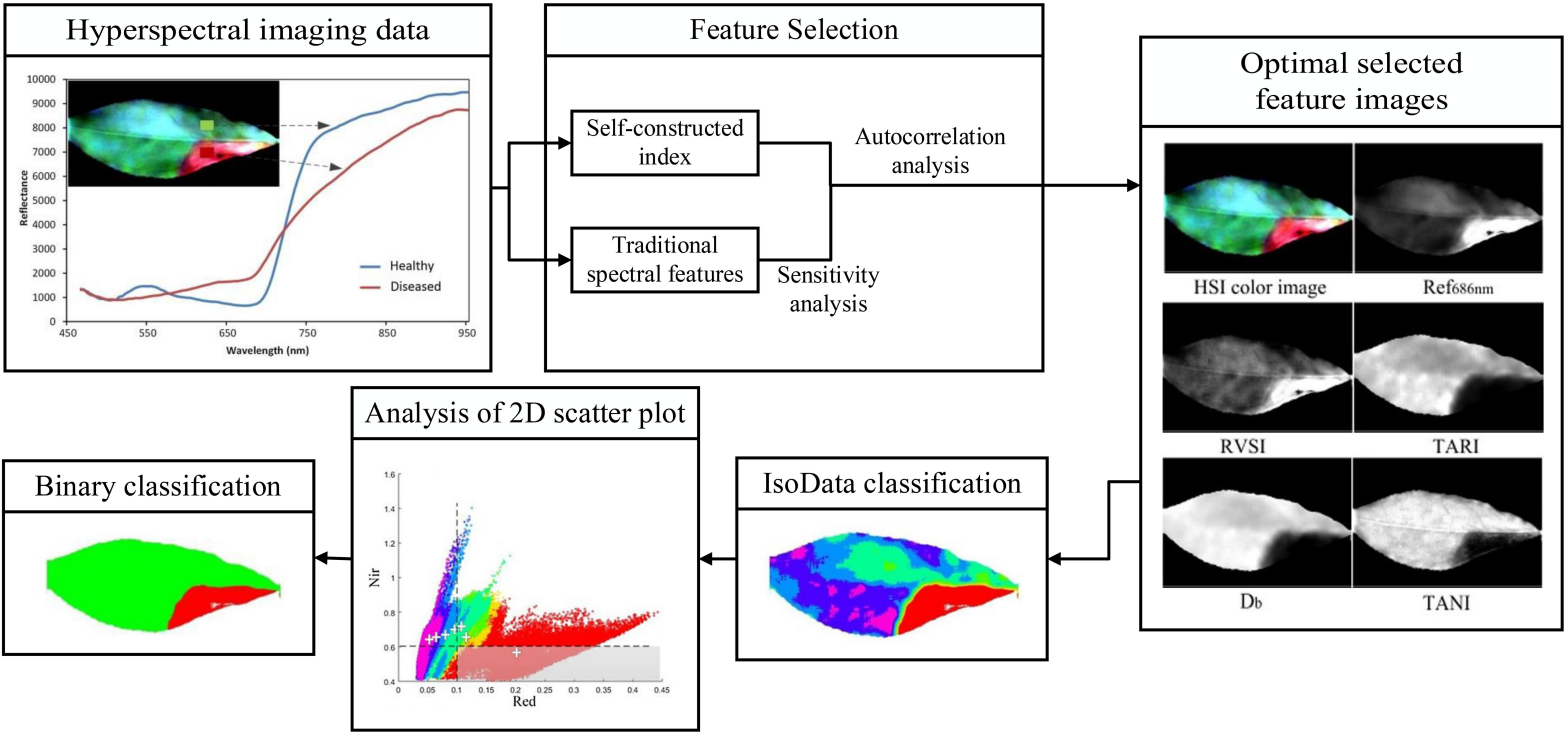
Schematic of the workflow for detection of anthracnose in tea plants based on hyperspectral imaging.

Biotic stress refers to the attack and interference of biological factors such as pathogenic microorganisms, pests, weeds, or other plant competition to which the tea plant is subjected during the growth process (Jeyaraj et al., 2020; Zahir et al., 2022). For example, hyperspectral technology was applied to detect anthracnose, a common foliar disease on tea plants. As shown in **Figure 5**, Yuan et al. screened the optimal hyperspectral feature set suitable for anthracnose scab detection by observing the red-shift phenomenon due to anthracnose in the wavelength range of 450-950 nm, and further used autocorrelation analysis to accurately and rapidly identify the anthracnose scab region on tea leaves. They combined unsupervised classification and two-dimensional threshold adaptation to construct an analytical framework for scab detection, which achieved an overall accuracy of 94% in recognizing the disease at the pixel level. Compared with traditional pixel-based classification methods, this method can effectively identify diseased tea leaves and analyze the degree of infection, indicating that hyperspectral technology can help improve tea plant disease detection and field management (Yuan et al., 2019). In the discrimination of leaf diseases with multiple similar symptoms, Lu et al. used hyperspectral technology (420-946nm) for identifying and distinguishing between white star disease and anthracnose that have similar image features. The highly similar morphological characteristics in mean spectra of the whole leaf infected by the two diseases resulted in relatively low data separability, while the mean spectra of the diseased spot regions were significantly different. Therefore, the mean spectra of diseased spot areas extracted after threshold segmentation and mask processing were combined with different machine learning models for classification, and the results of the study showed that the prediction accuracy of the ELM model based on the neural network structure was higher than that of the SVM model using different kernel functions, and its classification accuracy reached 95.77% (Bing et al., 2021). For these two similar diseases, hyperspectral technology can accurately recognize and detect the disease severity at the early stage of the onset of tea plant diseases. Compared to the computer vision technology, the above studies show that hyperspectral technology has certain advantages in the early detection of tea plant diseases, however its application is still mainly based on the external characteristics of tea plants affected by diseases, and it lacks the ability to qualitatively detect tea plants at the early stage of infection by pathogens. Therefore, further researches should take the implementation of predictive analysis in the early infect stage of tea plant disease into consideration, so as to realize the effective prevention and control of tea plant diseases (Lowe et al., 2017).

To minimize the loss caused by pests, pests early detection plays a key role in preventing the spread of pests. In the actual tea garden environment, the combined presence of pests and diseases is a common phenomenon, and it is often difficult to discriminate between plants diseases and pests due to the similarity of their damage symptoms. Cui et al. proposed a method based on canopy hyperspectral data (450-950nm) for detecting and distinguishing between three tea plant stresses with similar external characteristics (anthracnose, tea leafhopper and sunburn). Through spectral sensitivity analysis, the best spectral features in the three processes of removing plant background, identifying plant damaged areas and distinguishing tea plant stresses were determined, and then K-means clustering and KNN were used to construct a model for detecting damaged areas of the tea plant, and finally Fisher linear discriminant method was used to discriminate the stress types in the damaged areas. The validation results of the model showed that the detection accuracy of the damaged areas could reach 95% and the accuracy for stress discrimination was 98% (Cui et al., 2021). As can be seen from the above studies, the research method based on hyperspectral imaging data mainly establishes the relationship between spectral features and the degree of disease or pest types through various statistical discrimination or data mining algorithms, which is similar to the analysis method of the process of identifying the disease of tea plant, and thus reaches an accurate analysis of the infected areas under different types of stresses, revealing the potential application in large-scale tea plantation disease management, particularly in real-time monitoring and accurate diagnosis. By reducing pesticide usage and optimizing pest control strategies, hyperspectral imaging technology can significantly mitigate the negative environmental impact of agricultural production while enhancing both the yield and quality of tea leaves.

#### 4.1.2 Abiotic stress

During the initial period when tea plants are in abiotic stress, tea plants often do not exhibit obvious external characteristic changes. However, significant external trait differences may emerge only when the stress reaches a certain severity level, at which point the stress may have caused irreversible damage to the tea plants. Therefore, early identification and evaluation of characteristic changes in tea plants under abiotic stress are vital for devising timely measures to mitigate or reverse the stress effects (Gerhards et al., 2019; Krishnatreya et al., 2021). As early signs of abiotic stress in tea plants are usually reflected by changes in relevant biochemical parameters within the tea plant tissues during the stress response process, these changes can be captured as corresponding spectral differences. This makes it possible to qualitatively analyze the adaptation level of tea plants to current abiotic stresses. Nowadays, the application of hyperspectral technology to detect abiotic stress levels is being carried out and in-depth research. The following sections will discuss the three considered abiotic stress factors for tea plants: drought, low temperature, and heavy metal.

##### 4.1.2.1 Drought stress

With the global climate warming leading to an increased frequency of extreme weather events and the lack of agricultural water causing ineffective irrigation of tea plants, tea plants are facing more frequent drought stress, negatively impacting their growth, development, and yield. To combat the adverse effects caused by drought stress, tea plants undergo a series of physiological defense mechanisms, including stomatal regulation, osmotic adjustment, and regulation of the antioxidative enzyme system (Liu and Chen, 2014; Tony et al., 2016). Hyperspectral technology was used to understand the changes in physiological and biochemical parameters within these mechanisms to assess the severity of drought stress. Chen et al. obtained five drought-related physiological and biochemical index parameters, namely malondialdehyde (MDA), electrolyte leakage (EL), maximal efficiency of photosystem II (Fv/Fm), soluble saccharide (SS), and drought damage degree (DDD), during the drought stress treatments with different levels in tea seedlings. By trying various data processing algorithms and modeling methods, they predicted the drought stress severity for different tea seedlings. Among these, UVE-SVM detection model for the drought damage degree (DDD) achieved a high prediction accuracy (Rcal=0.97, Rp=0.95, RPD=4.28), providing a comprehensive and objective assessment of the tea plants’ drought resistance (Chen et al., 2021).

In another study by the same research team, hyperspectral imaging technology (400-1000nm) was used to monitor drought stress in 10 different tea germplasm resources to determine their drought tolerance, proving the feasibility and effectiveness of hyperspectral technology in screening for drought-resistant tea plant germplasm. 3 indicators (malondialdehyde, soluble sugar, and total phenol) directly used in drought evaluation positively contributed to constructing a comprehensive index, DTC (Drought Tolerance Coefficient), for assessing the drought tolerance of tea plants. The results indicated that the model constructed with the MSC-2D-UVE-SVM method showed the best performance in screening for drought-resistant tea germplasm resources ( ${\text{R}}^{2}$ =0.77, RMSE=0.073, MAPE=0.16) (Chen et al., 2022). Based on the above research, tea plant drought stress detection using hyperspectral technology, from the initial stages of genetic selection to the entire cycle of plant life activities, have acquired real-time precise monitoring technologies for traits exhibited by tea plants under drought stress. However, the tea plants’ growing environments and their growth stages causes variability in their drought stress responses to some extent. Therefore, further research into tea plants with complex phenotypic trait changes must be conducted, and these studies will bring greater prospects for improvement in tea plant breeding optimization and precision cultivation. In general, current research on environmental stress detection in tea plants predominantly focuses on single types of stress, overlooking the reality that tea plants often encounter multiple environmental stresses simultaneously in their natural growth processes (Safavi-Rizi et al., 2021). For instance, tea plants might concurrently endure the dual stresses of drought and high temperatures during the same period, a scenario in which their phenotypic responses could markedly differ from those observed under singular conditions of drought or heat stress alone (Tang et al., 2023). This focus could severely limit the applicability of research findings, leading to an incomplete understanding of the overall tea plant phenotype and an inability to reveal the complex interplay and comprehensive responses of environmental factors that influence the growth and physiology of tea plants (Cao et al., 2015).

##### 4.1.2.2 Low temperature stress

Low temperature environment is one of the important constraints on the growth and development rate, tea yield and natural geographic distribution of tea plants. Under low temperature stress, the cell membrane structure and function of the tea plant will be damaged, triggering a series of abnormal physiological and biochemical reactions, leading to cellular metabolic disorders, and thus causing damage to the tea plant. Relevant studies have shown that tea plant produces protective enzymes such as catalase (CAT), peroxidase (POD), and superoxide dismutase (SOD) to scavenge excessive active oxygen free radicals accumulated under low temperature stress conditions (Wang et al., 2021). Therefore, changes in these protective enzymes can be tracked using hyperspectral techniques to measure the extent of cold stress.

In addition, some physiological indicators such as malondialdehyde, soluble proteins, and soluble sugars involved in the cold resistance response can also be used as dependent factors for assessing the degree of low temperature stress suffered by tea plants. Mao et al. investigated the spectral changes of tea leaves after exposure to low temperature stress at 8h and 12h intervals under normal (25°C), chilling (4°C), and freezing (-4°C) conditions. Various variable selection methods and machine learning models were used to analyze the contents of chlorophyll (SPAD value), soluble sugar (SS), and malondialdehyde (MDA), as well as the activities of catalase (CAT), peroxidase (POD), superoxide dismutase (SOD), and the Low Temperature Response Index (LTRI). The UVE-CNN model demonstrated high predictive accuracy corresponding to LTRI, with ${\text{Rp}}^{2}$ = 0.8631 and RMSEP = 0.325 (Mao et al., 2023a). This study identified the potential advantages of applying hyperspectral techniques to detect low temperature stress in tea plants, which could provide accurate data support for preventing damage caused by frost damage in tea plants. The multi-parameters evaluation model was generally superior to the single physicochemical index model in analyzing abiotic stress in tea tree. This may be due to the multi-parameters evaluation model in tea plant can be as internal standards of each other and their response to low temperature stress were considered. However, the selection of biochemical components in tea plant, and the mechanism between them and low temperature stress may need further investigation.

##### 4.1.2.3 Heavy metal stress

Heavy metals in tea plant mainly originate from anthropogenic factors, such as industrial emissions and excessive use of agricultural fertilizers, which cause pollution of air, water and soil. These heavy metals mainly enter the tea plant through two pathways, root absorption and foliar infiltration, leading to impaired nutrient absorption and metabolic function, and hindering the normal growth and development of the tea plant (Zhang et al., 2018; Sanaeifar et al., 2022). In addition, heavy metals may accumulate in tea leaves, posing a serious threat to human health. Therefore, timely monitoring and management of heavy metal pollution is crucial for tea plantations. Like other abiotic stresses, some stress-related physiological indexes of tea plants under heavy metal stress also changed with the progress of stress.

Jin et al. proposed a method using hyperspectral imaging (420-946 nm) combined with chemometrics to quantify the changes of four relevant indicators, chlorophyll (Chl), ascorbic acid (ASA), glutathione (GSH)and soluble protein (SP), in the foliage and roots of tea plant at different times and under different lead stresses. The quantitative analysis model for each indicator was established by optimal pretreatment and selection of characteristic wavelengths. All the models based on MLR were simple and efficient while showing good predictive effects, and the model prediction coefficient of determination ${\text{Rp}}^{2}\;$ could reach about 0.6~0.9 (Jin, 2019). Similarly, Sanaeifar et al. successfully applied a PLS-RBFNN model based on visible and near-infrared (Vis-NIR) spectroscopy to precisely predict the quality indices of tea leaves under foliar stress at different intervals and varying treatment levels (Sanaeifar et al., 2022). Both methodologies offer an effective approach for the rapid analysis and accurate detection of the physiological status of tea plant under heavy metal stress. However, the problem of heavy metal contamination from air and soil that tea plant may face is not limited to a single heavy metal, and there are differences in the effects of different heavy metals on tea trees. Therefore, the detection of various heavy metal stresses has the potential to become a valuable research direction in the field of hyperspectral technology-based detection of tea plant phenotypic information (Zhang et al., 2021).

### 4.2 Growth and developmental information monitor

Information on the growth and development of the tea plant mainly includes water, photosynthetic pigments, nutrient elements, and morphological and structural parameters of the tea plant. This information can be used to gain an in-depth understanding of the real-time physiological state of the tea tree and the complexities of the long-term growth process, which in turn provides a scientific basis for assessing and improving tea yields (Li et al., 2022). The application of hyperspectral technology allows for more accurate quantitative analysis and visual representation of the phenotypic parameters to be measured in a non-destructive manner.

Water is the most basic life element of the tea plant, which is directly involved in the growth and development of the tea plant, physiological metabolism and the construction of morphological structure. Meanwhile, water also plays an important regulating role in the formation of tea quality components. Therefore, real-time and accurate detection of tea plant water status is of great significance in tea plant cultivation and tea garden irrigation. Estimating the moisture content of tea plant leaves has been an important area of research in hyperspectral technology (Zhang et al., 2018). Researchers have used near-infrared hyperspectral imaging in conjunction with a variety of combinatorial algorithms for quantitative modeling and visualization of tea leaf moisture distribution (Sun et al., 2019). In addition, Wei et al. considered that the hyperspectral data (380-1030nm) of the front and back sides of the tea leaves would be different during the tea production process, and designed a logistic classification regressor with a 100% correct classification rate to identify the front and back sides of the tea leaves, and then used the characteristic spectral bands corresponding to the front and back sides of the tea leaves to build a Least Squares Support Vector Machine (LS-SVR) model to generate tea leaf water content distribution maps (Wei et al., 2019). These published studies have mainly focused on the accurate analysis of tea plant water content at the leaf scale. In order to map the overall moisture status of tea trees more comprehensively, future studies should aim to extend the assessment to the canopy level of tea plants. Research in the field of monitoring the water status of other crops, such as the studies by Lü et al., has demonstrated the potential of canopy hyperspectral imaging technology to quantify and predict the water status of Arabidopsis thaliana (Lü et al., 2020). Similarly, Wocher et al. have shown a significant relationship between canopy reflectance and moisture content in winter wheat and maize (Wocher et al., 2018). These studies underscore the effective application of spectral data at the canopy scale across different crops, providing crucial foundations and references for extending this approach to monitoring the water status of tea plants.

The photosynthetic pigments in the tea plant are mainly composed of chlorophyll and carotenoid, which are important components of photosynthesis. The content of photosynthetic pigments directly reflects the photosynthetic capacity of tea plants and the degree of environmental adaptation. At present, hyperspectral technology has become an effective tool for accurately estimating the content of photosynthetic pigments and their spatial distribution in tea tree leaves. For example, Zhao et al. successfully calculated the chlorophyll concentration at the scale of image elements by the MSAVI2 (Modified secondary soil adjustment vegetation index) prediction model (Zhao et al., 2011). Meanwhile, the joint measurement of chlorophyll and carotenoid content has the potential to detect environmental stresses. Sonobe et al. used the KELM (Kernel-based Extreme Learning Machine) model to establish the relationship between hyperspectral data (400-900nm) and the content of the three photosynthetic pigments (chlorophylls a, b, and carotenoids), demonstrating their accuracy, with root mean square errors of 1.95 ± 0.36, 1.08 ± 0.11 and 0.68 ± 0.10 $\text{µg}/{\text{cm}}^{2}$ for each photosynthetic pigment corresponding model, respectively (Sonobe et al., 2018a). In addition, nitrogen in tea plant leaves mainly exists in photosynthetic pigments, so the nitrogen nutritional status of tea trees can be effectively monitored by grasping the dynamic changes of photosynthetic pigment content in real time. Using hyperspectral imaging technology, through the SNV-PLSR model constructed on the basis of the entire wavelength range of 400-1000 nm and the 2-Der-PLSR model constructed on the basis of characteristic wavelengths, Wang et al. highly accurate prediction of chlorophyll a, chlorophyll b, total chlorophyll and carotenoids contents in tea leaves under different nitrogen application levels was successfully realized (Wang et al., 2019). This series of studies vividly demonstrates the high precision and efficiency of hyperspectral technology in measuring photosynthetic pigments in tea plants. The dynamic changes in plant photosynthetic pigments are closely linked to their physiological state. Subsequent research can translate the spatiotemporal dynamics of photosynthetic pigments into quantitative assessments of key indicators for tea plant growth and development, thereby expanding its potential applications in actual tea production.

**Figure 6:**
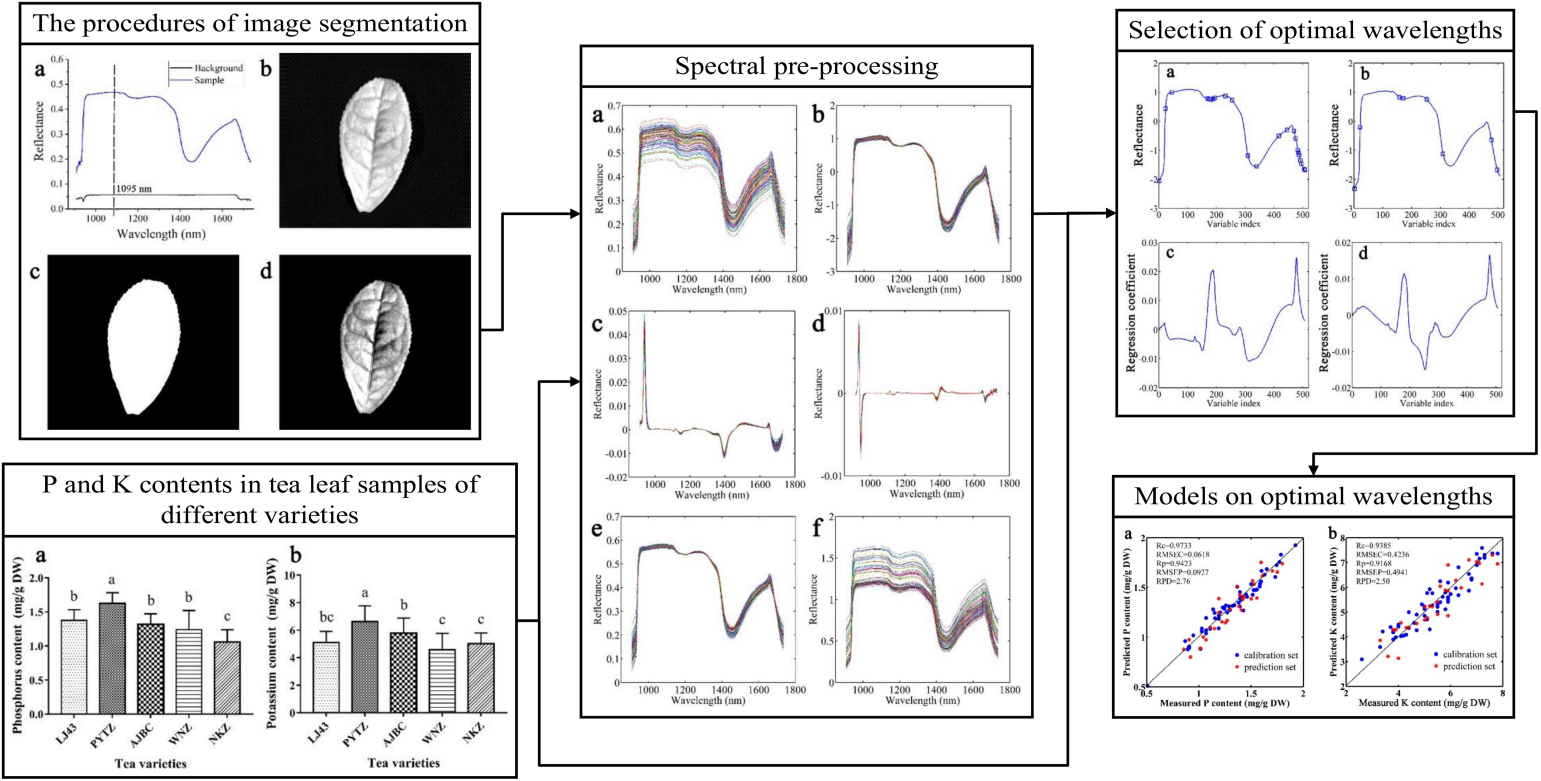
Illustration of the image processing and data analysis process used for quantifying the phosphorus and potassium content in different tea plant cultivars.

Nitrogen, phosphorus and potassium are essential nutrients for the growth, development and yield formation of tea plants, and they usually supply in the form of fertilizers to tea plants. Efficient, non-destructive, and accurate monitoring of the nutrient status of tea trees can help to diagnose the growth status of tea plants, improve the efficiency of fertilizer application, and reduce the damage of over-fertilization to soil nutrients and the negative impact on the environment, so as to realize the precision management of tea plant cultivation. Wang et al. utilized hyperspectral technology combined with a multivariate classification algorithm to discriminate the nitrogen application level of tea trees. The study firstly extracted the five optimal wavelengths by principal component analysis from the hyperspectral data containing 553 wavelengths in the spectral range of 400-1000 nm, and then extracted texture features from the images of the optimal wavelengths by using the gray level gradient covariance matrix (GLGCM). Finally, the SVM model using fused data gave the best performance with highest correct classification rate of 100% for prediction set (Wang et al., 2018). In a related study, the research team combined hyperspectral image technology (400-1000nm) with chemometrics methods to accurately discriminate the nitrogen level and status of tea plants by using the SNV-LS-SVM model, and its correct classification rate reached 82% and 92%, respectively. Meanwhile, the nitrogen content of tea tree leaves was successfully quantified by the SNV-PLSR model with a correlation coefficient Rp of 0.924 (Wang et al., 2020c). In addition, the research team extended its research results to the determination of phosphorus and potassium content in tea leaves (**Figure 6**), and successfully achieved high-precision prediction results of phosphorus and potassium content in tea samples of different varieties with correlation coefficients Rp of 0.9423 and 0.9168, respectively, by the SPA-MLR prediction model (Wang et al., 2020a). This series of studies have demonstrated the promising application of hyperspectral technology in dynamic nutrient tracking and field fertilization management of tea trees, but there are many challenges and limitations to be faced in translating the results of the studies into practical applications. One of the common problems is the weak transferability of the constructed prediction models, which mainly stems from the influence of different tea plant breeds, geographical locations and environmental factors on the prediction of tea tree growth and development information. Therefore, diagnostic models with broader applicability by relying on hyperspectral data of different tea tree species under various environmental conditions should be built in the future.

Morphological and structural parameters of tea tree are also an important topic in the study of tea plant growth information detection, which mainly involves leaf area, biomass and crown width and other related parameters. Various types of vegetation indices derived from hyperspectral data can be used to monitor the changes of morphological and structural parameters, effectively assess the growth status of tea plant, and reflect the production potential of tea plant. To address the problems of time-consuming and destructive in the traditional manual detection methods of tea tree LAI (leaf area index), Wu et al. analyzed the correlation between LAI and hyperspectral feature variables, screened out the three feature variables with the highest correlation (amplitude of yellow edge, green peak reflectance and red valley position), and set up different inversion models through the three variables, among which the logarithmic fitting model with green peak reflectance ${\text{R}}_{\text{g}}$ as the independent variable was modeled better with validation sample ${\text{R}}^{2}$ and RMSE values of 0.764 and 0.0876, respectively. This study verified the feasibility of applying hyperspectral technology for real-time, rapid and non-destructive measurement of tea tree leaf area index method (Wu et al., 2018). As the morphological and structural characteristics of tea tree are more intuitive in observation, conventional spectral detection techniques can already provide more accurate results. Hyperspectral techniques have not generated a strong demand in the study of tea tree morphological structural parameters due to their high economic cost and complexity of analyzing and processing the data. However, through an in-depth analysis of hyperspectral data, its rich information across spectral bands and high-resolution spectral features can compensate for the limitations of traditional methods in terms of vegetation spatial resolution and data sensitivity, enabling the construction of vegetation indices capable of accurately detecting morphological structural parameters in remote sensing.

### 4.3 Quality indicator detection

The quality of tea is one of the key factors determining its market value and processing methods. The content of organic chemicals in tea plant leaf, such as tea polyphenols, amino acids and caffeine, is an important indicator of tea quality and flavor (Peng et al., 2016; Cao et al., 2020). Traditionally, the content of these organic chemicals has been assessed by destructive sampling followed by wet chemical methods, which are time-consuming and labor-intensive and difficult to adequately represent spatial variability in whole tea plant. Dutta et al. analyzed *in situ*-measured hyperspectral data in the range of 347-2506 nm by applying different multivariate analytical methods to screen the best predictive models, and developed a partial least squares regression (FDR-PLSR) model for first-order derivative spectra to achieve accurate estimation of tea polyphenol content at the leaf level, with ${\text{R}}^{2}$ and RMSE values of 0.81 and 1.39 for the model, respectively (Dutta et al., 2015). Hyperspectral techniques also offer the possibility of estimating and monitoring the quality of tea on a spatial scale. Tu et al. accurately classified tea tree varieties in large-scale tea plantations based on the spectral characteristics of tea tree canopy data acquired by a 450-998 nm hyperspectral camera mounted on a UAV. The hyperspectral data were also processed through partial least squares regression (PLSR) using standard normal variable transformation (SNV) preprocessing method to predict tea polyphenol and amino acid contents, although the model showed a relatively significant prediction accuracy ( ${\text{R}}_{\text{CV}}$ = 0.66, ${\text{RMSE}}_{\text{CV}}$ = 13.27) in terms of the ratio of tea polyphenols to amino acids (the main indicator of tea sensory quality), which proves that there is an association between the spectral data and quality indicators. To apply this correlation to the prediction of actual tea tree quality indicators based on the UAV platform, more in-depth research and exploration are still needed to improve the accuracy of the model (Tu et al., 2018). On the basis of existing researches, hyperspectral technology can be used to achieve independent analysis and measurement of multiple subcomponent contents of the main components of tea, in order to accurately feedback the current quality of tea and provide favorable guidance for the optimization of the subsequent production and processing processes. On the other hand, current research primarily relies on spectral data obtained in a single instance, without considering the temporal dynamics of tea plant phenotypic characteristics throughout phenological cycles (Mahlein et al., 2018). Phenotypic expression in tea plants may vary over time, necessitating continuous observation to accurately capture these dynamics. It is recommended that future studies expand the range of tea plant sample collection to encompass phenotypic differences in different environments. By conducting long-term observational studies to establish comprehensive tea plant phenotypic databases, the statistical significance and reliability of research can be enhanced, facilitating the development of more refined and generalizable models (Wijewardane et al., 2023).

## 5 Summary and outlook

In this paper, we comprehensively review the research progress of hyperspectral imaging technology in the field of tea plant phenotyping information detection, the main applications of which involve environmental stress diagnosis, growth and development information monitoring, and quality indicator detection of tea plant. Hyperspectral technology in the detection of tea plant phenotypic information is still in its nascent stage, with notable gaps in research. Despite the considerable enrichment of our understanding of plant phenotypes facilitated by this technique, the issue of potentially missed critical phenotypic information due to restricted acquisition at specific wavelengths or resolutions remains pertinent. It thus becomes markedly important to introduce more advanced hyperspectral technologies to enhance the accuracy and comprehensiveness of data collection. In the data processing and analysis phase, hyperspectral data necessitates navigation through a complex and meticulous data processing workflow. Errors occurring at any stage within this workflow harbor the potential to precipitate data inconsistencies. Consequently, the development of more efficacious data processing and analysis algorithms is instrumental in extracting more precise and reliable information from hyperspectral data. In addition, in order to ensure the comparability of data between different studies, it is of great significance to further establish standardized hyperspectral data processing and analysis methods. In application, current research on tea plant phenotypic detection also exhibits certain limitations, particularly the absence of a comprehensive method that spans multiple observational scales, involving leaf organs, plants individual, and populations. Integrating observations from multiple scales is crucial for a comprehensive understanding of the phenotypic information of tea plants. However, a unified methodological framework to integrate findings across different scales has yet to be established. In addition, future research should focus on the cross-stress response of tea plant, in order to deconstruct the association between phenotypic characteristics of tea tree in a more comprehensive perspective, and thus promote the deep optimization of tea plant breeding strategies and cultivation management.

## Author contributions

BL: Writing – review & editing. HS: Project administration, Writing – original draft. LZ: Writing – review & editing. FC: Conceptualization, Methodology, Writing – review & editing. KW: Writing – review & editing.
